# Two novel missense substitutions in the *VSX1* gene: clinical and genetic analysis of families with Keratoconus from India

**DOI:** 10.1186/s12881-015-0178-x

**Published:** 2015-05-12

**Authors:** Rohit Shetty, Rudy M.M.A. Nuijts, Soumya Ganesh Nanaiah, Venkata Ramana Anandula, Arkasubhra Ghosh, Chaitra Jayadev, Natasha Pahuja, Govindasamy Kumaramanickavel, Jeyabalan Nallathambi

**Affiliations:** Cornea and Refractive Surgery Department, Narayana Nethralaya Postgraduate Institute of Ophthalmology, Bangalore, India; Cornea Clinic, Department of Ophthalmology, Maastricht University Medical Center, 6211 LK Maastricht, The Netherlands; GROW Research Laboratory, Narayana Nethralaya Foundation, Bangalore, India

**Keywords:** Visual system homeobox gene (*VSX1*), Familial keratoconus, Mutation screening, CVC (Chx10/Vsx–1 and ceh–10) domain, *In sillico* analysis, Missense mutation, Haplotype analysis

## Abstract

**Background:**

Visual system homeobox gene (*VSX1*) plays a major role in the early development of craniofacial and ocular tissues including cone opsin gene in the human retina. To date, few disease-causing mutations of *VSX1* have been linked to familial and sporadic keratoconus (KC) in humans. In this study, we describe the clinical features and screening for *VSX1* gene in families with KC from India.

**Methods:**

Clinical data and genomic DNA were collected from patients with clinically diagnosed KC and their family members. The study was conducted on 20 subjects of eight families from India. The coding exons of *VSX1* gene were amplified using PCR and amplicons were analyzed by direct sequencing. Predictive effect of the mutations was performed using Polyphen-2, SIFT and mutation assessor algorithms. Additionally, haplotypes of *VSX1* gene were constructed for affected and unaffected individuals using SNPs*.*

**Results:**

In the coding region of VSX1, one novel missense heterozygous change (p.Leu268His) was identified in five KC patients from two unrelated families. Another family of three members had a novel heterozygous change (p.Ser251Thr). These variants co-segregated with the disease phenotype in all affected individuals but not in the unaffected family members and 105 normal controls. *In silico* analysis suggested that p.Leu268His could have a deleterious effect on the protein coded by VSX1, while p.Ser251Thr has a neutral effect on the functional properties of VSX1. Haplotype examination revealed common SNPs around the missense change (p.Leu268His) in two unrelated KC families.

**Conclusions:**

In this study, we add p.Leu268His, a novel missense variation in the coding region of VSX1 to the existing repertoire of *VSX1* coding variations observed in Indian patients with the characteristic phenotype of KC. The variant p.Ser251Thr might be a benign polymorphism, but further biophysical studies are necessary to evaluate its molecular mechanism. The shared haplotype by two families with the same variant suggests the possibility of a founder effect, which requires further elucidation. We suggest that p.Leu268His might be involved in the pathogenesis of KC, which may help in the genetic counselling of patients and their family.

## Background

Keratoconus (KC: OMIM 14830) is a progressive ectatic disorder of the cornea characterized by thinning of the central cornea leading to protrusion and progressive, irregular astigmatism. Though there are several treatment modalities available, severe KC remains an indication for corneal transplantation [1, 2]. The mean age of onset is 15.39 years with a prevalence of 0.0003 %–2.3 % that affects both genders and all ethnicities across the globe [3]. The disease is a complex heterogeneous disorder with risk factors like chronic eye rubbing and atopy playing a significant role besides ultraviolet light induced oxidative stress [4–6]. The genetic basis for keratoconus has always been an accepted theory considering its familial occurrence and high concordance in monozygotic twins [4, 7, 8]. Though most KC cases are sporadic, it has been noted that 6–10 % of cases have a positive family history [9, 10]. Inheritance in KC can be dominant or recessive; with autosomal dominant inheritance, the disease exhibits variable phenotypes with incomplete penetrance [9].

Linkage analysis has identified several genomic loci in KC families thereby establishing genetic heterogeneity [11–15]. Genes with mutations (*VSX1*, *DOCK9, TGFβI, SOD1, FLG, ZEB1*) were found to be responsible for only a small fraction of KC cases in select populations around the world [16–21]. Nevertheless, *VSX1* mutations have been identified in two different corneal phenotypes - posterior polymorphous corneal dystrophy (PPCD) and KC [16]. Genetic analysis of KC patients from different ethnic backgrounds has revealed several coding variations in the *VSX1* gene [22–26]. So far, four pathogenic *VSX1* mutations have been reported in the KC phenotype. Hence, the significance of a genetic basis for KC is still unclear [22–26]. VSX1 is a paired-like homeodomain transcriptional factor gene localized in 20p11.21. It is expressed in the adult cornea and adult retinal cDNA libraries [27], inner nuclear layer of the human retina and embryonic craniofacial tissue [28]. The human *VSX1* gene has five exons that encodes for a 365–amino acid protein with homeobox DNA binding domain and a CVC (Chx10/Vsx–1 and ceh–10) domain, which is highly conserved among vertebrates. In this present study, we correlate the genetic, and clinical features of KC patients and their families of Indian origin with *VSX1* gene variants.

## Methods

### Study subjects and clinical examination

Twenty affected individuals from eight unrelated KC families, 11 unaffected family members, and 105 ethnically matched normal controls were included in this study. All patients were examined at the Cornea and Refractive Surgery Department, Narayana Nethralaya Postgraduate Institute of Ophthalmology, Bangalore, India. The study followed the tenets of the Declaration of Helsinki and was approved by the Institutional Ethical Committee (IEC–C/2013/07/01). All patients underwent visual acuity assessment, a detailed slit lamp examination with topographic and pachymetric evaluation on the Pentacam HR (Oculus Inc.) and Orbscan (Orbtek, Baush, & Lomb). Keratoconus was graded according to the Amsler-Krumeich Classification [29]. If KC was detected in more than one member of the family, the entire family was counselled, detailed informed consent taken, and blood collected for genetic analysis.

### Genetic study

A detailed family history was taken including history of ocular and non-ocular hereditary disorders and pedigree charts drawn accordingly. The total genomic DNA was isolated from peripheral blood leukocytes by the salt precipitation method [30] from all study subjects. For mutational analysis, the entire coding exons of *VSX1* and their flanking intronic junctions were amplified by PCR in eight probands using the primer reported elsewhere [25]. The PCR products were sequenced on 3130xl Genetic Analyzer (Applied Biosystems) according to the manufacturer’s protocol. Sequencing results were analyzed in chromatogram viewer (FinchTV 1.40), pairwise BLAST (Basic Local Alignment Search Tool) [31] to examine if there were any changes from the normal *VSX1* sequence available in the database (NM_014588, ENSG00000100987). The segregation of nucleotide changes were analysed in eight affected and four unaffected individuals from three unrelated families by direct sequencing method. In addition, exon 4 of *VSX1* was sequenced in 105 unrelated ethnically matched normal controls to validate the pathogenicity of nucleotide variations.

### Bioinformatics analysis

In order to predict the effect of nucleotide change at the protein level, we used *in silico* prediction servers Polyphen–2 (http://genetics.bwh.harvard.edu/pph2/), SIFT (http://sift.bii.a-star.edu.sg), Mutation Assessor (http://mutationassessor.org/v1/) and PROVEAN (http://provean.jcvi.org/index.php). Clustal Omega (https://www.ebi.ac.uk/Tools/msa/clustalo/) and multiple sequence alignment programs were used to check the evolutionary conservation of VSX1 protein in other vertebrates. The effect of amino acid changes in the stability of VSX1 was assessed by using the MUpro (version 1.0, http://mupro.proteomics.ics.uci.edu/) prediction server (AAMSPSM).

### Haplotype analysis

To examine the disease and mutation associated haplotypes of the eight affected and four unaffected individuals from two unrelated KC families, we analysed four intragenic SNPs (rs12480307, rs6138482, rs56157240), and (IVS3–24C > T) flanking *VSX1* by direct sequencing*.* Haplotypes were constructed manually.

## Results

In this study, we analysed 20 affected individuals of eight families with a clinical diagnosis of KC for mutations in the *VSX1* gene (Table 1). A novel missense coding variant (p. Leu268His) was found in five patients from two unrelated KC families (KC_01,KC_02). Another novel heterozygous missense change (p. Ser251Thr) was identified in a third KC family (KC_03) with two affected siblings and their affected father. The clinical features of the affected individuals are summarized in Table 2.

**Table 1:** Summary of genotype and phenotype characteristics in the study subjects

Family ID	Individuals	Age at diagnosis/Sex	Keratoconus	Segregation of *VSX1* nucleotide changes	
				Coding variants	SNPs
**KC–01**	I:1	40/F	No	-	rs56157240,
	I:2	35/M	Yes/RE	L268H	rs12480307, rs56157240, IVS3–24C > T
	II:1	22/M	Yes/RE	L268H	rs12480307, rs56157240, IVS3–24C > T
	II:2^a^	19/M	Yes/BE	L268H	rs12480307, rs56157240
**KC–02**	I:1	42/F	No	-	rs12480307, rs6138482, IVS3–24C > T
	I:2	37/M	Yes/LE	L268H	rs12480307, rs56157240, rs6138482,
	II:1^a^	16/M	Yes/BE	L268H	rs12480307, rs56157240, rs6138482,
	II:2	12/F	No	-	rs12480307,
**KC–03**	I:1	45/F	No	-	rs56157240, rs6138482
	I:2	50/M	Yes/BE	S251T	IVS3–24C > T
	II:1	20/F	Yes/BE	S251T	IVS3–24C > T
	II:2^a^	18/M	Yes/BE	S251T	rs56157240
**KC–04**	I:1	53/M	No	-	rs12480307, rs56157240
	I:2	44/F	Yes/BE	-	IVS3–24C > T
	II:1^a^	29/M	Yes/LE	-	rs56157240
	II:2	26/F	No	-	rs56157240, IVS3–24C > T
**KC–05**	I:1	60/M	No	-	rs6138482, rs56157240, IVS3–24C > T
	I:2	47/M	Yes/LE	-	rs56157240,
	II:1	31/M	Yes/LE	-	IVS3–24C > T, rs6138482
	II:2^a^	27/F	Yes/LE	-	rs56157240, rs6138482
**KC–06**	I:1	43/F	Yes/BE	-	rs12480307, rs56157240,
	I:2	50/M	No	-	rs6138482
	II:1	29/M	No	-	rs12480307, rs6138482
	II:2^a^	24/F	Yes/BE	-	rs56157240, rs6138482
**KC–07**	I:1	53/F	Yes/BE	-	rs12480307, rs6138482, IVS3–24C > T
	I:2	61/M	No	-	rs12480307, rs6138482,
	II:1^a^	34/M	Yes/RE	-	rs6138482, IVS3–24C > T
**KC–08**	I:1	39/F	No	-	rs12480307, rs6138482, rs56157240
	I:2	45/M	Yes/RE	-	rs6138482, rs56157240
	II:1^a^	18/M	Yes/RE	-	rs12480307, rs56157240
	II:2	16/F	Yes/RE	-	rs6138482, rs56157240

RE: Right eye, LE: Left eye, BE: Both eye, M-Male, F-Female, The symbol (−) denotes the absence. Symbol (^a^) indicates the probands

**Table 2:** Clinical features of affected individuals from KC families with *VSX1* coding variants

Family ID	BCVA	Scisorrs’ retinoscopic reflex	Location of corneal thinning	Corneal topography	Thinnest	Fleisher's ring	Apical corneal scarring	KC
					Pachymetry			
					*(*μ*m)*			
KC–01_II:2	0.15/ 0.1	+	RE: Central	RE, LE: Infero-superior, asymmetry Inferior cone, steepening corresponds with anterior, and posterior elevation.	RE: 447	BE: +	RE: +	Both eyes
			LE: Inferior		LE : 450			
KC–01_II:1	0/ 0	+	RE: Central	RE: Infero-superior, asymmetry, Infero nasal cone.	RE: 482	RE: +	-	RE: +
			LE: Normal		LE: 493			LE: Normal
				LE: Normal				
KC–01_I:2	0.47/0	+	RE: Infero temporal cone	RE: Inferior-Superior, asymmetry, Infero temporal cone, central cone with skewing of axis of 40°	RE: 404	RE: +	-	RE: +
					LE: 440			LE: Normal
			LE: Normal					
				LE: Normal				
KC–02_II:1	0.15/0.15	-	RE: Central	RE: Posterior elevation	RE: 490	BE: +	-	Both eyes
			LE: Central	LE: Central corresponds with thinnest pachymetry and posterior elevation	LE: 414			
KC–02_I:2	0/ 0	-	RE: Normal	RE: Normal	RE: 506	-	-	RE: Normal
			LE: Infero- temporal	LE: Posterior elevation,	LE: 488			
				Infero-temporal cone				LE: +
KC–03_II:2	0.47/ 0.15	-	RE: Central	RE: Advanced KC,	RE: 333	+	-	Both eyes
			LE: Central	central cone with gross posterior elevation	LE: 443			
				LE: Central cone				
KC–03_II:1	0/0		RE: Central	RE: Posterior elevation,	RE: 526	-	-	Both eyes
			LE: Inferior	Central cone LE: Infero nasal cone	LE: 532			
KC–03_I:2	0.15/0.15	-	RE: Central	BE: Inferior cone, inferior- superior asymmetry with similar involvement in both eyes	RE: 419	-	-	Both eyes
			LE: Central		LE: 421			

RE: Right eye, LE: Left eye, BE: Both eye, BCVA- Best corrected visual acuity*,* KC-Keratoconus, The symbols + and - represent present and absent, respectively. M-Male, F-Female

### Mutation screening of *VSX1* gene

**Fig. 1:**
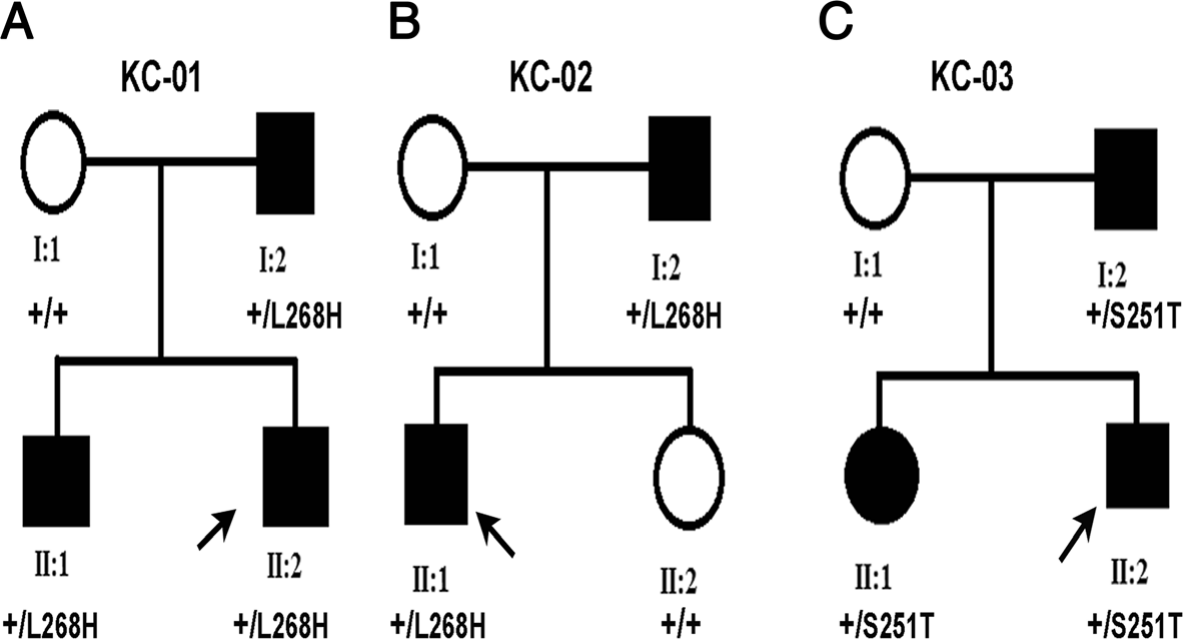
Pedigrees of the KC families with novel coding variants in *VSX1* Legend: **A**, **B**, **C** denotes three unrelated families. Squares and circles indicate males and females, respectively. Black symbols indicate affected members and open symbols indicate unaffected individuals. The black arrow indicates the proband, the sign ‘+’ represents the wild type and the mutations identified are p. Leu268His, p. Ser251Thr

**Fig. 2:**
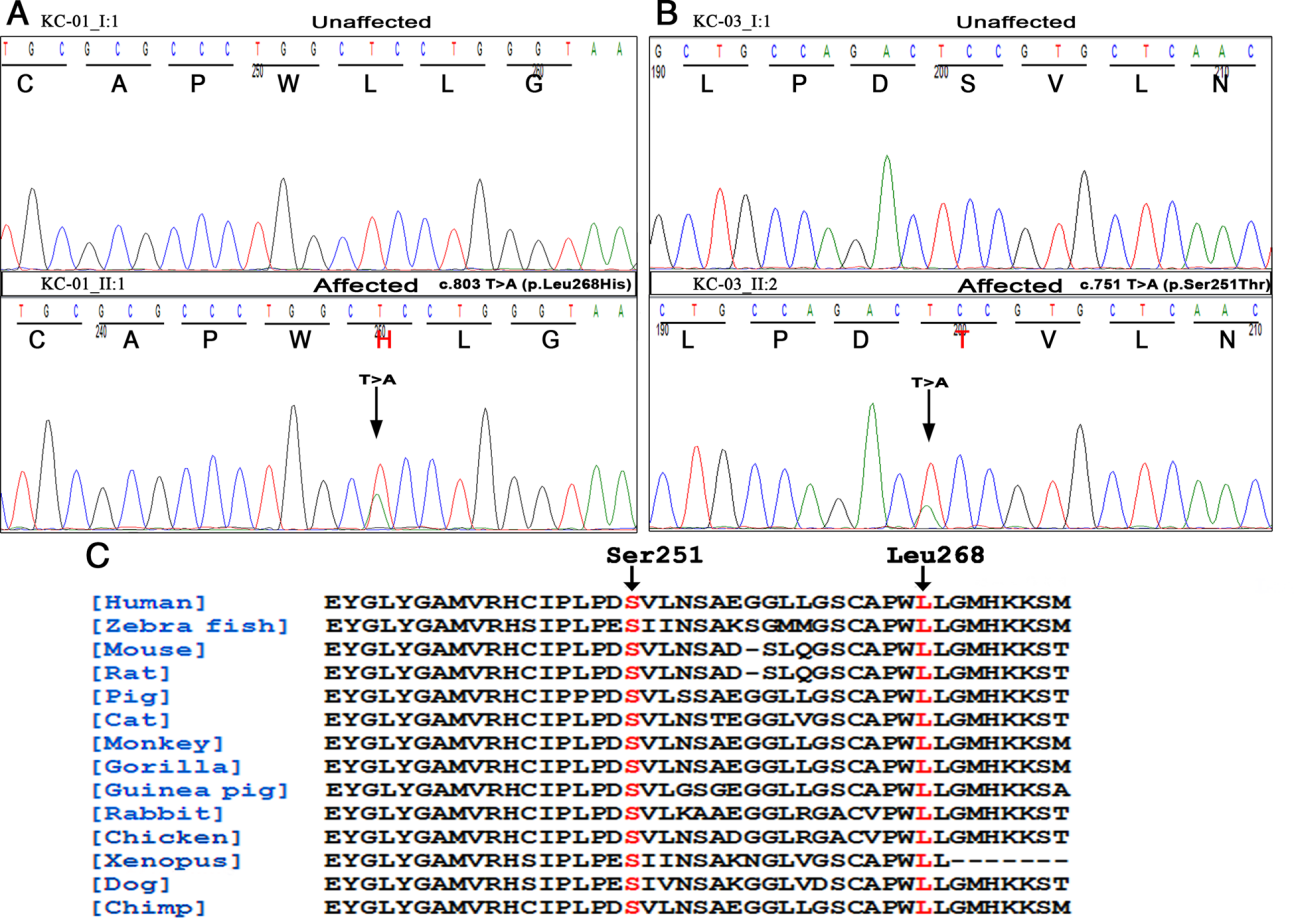
Novel *VSX1* coding variants in KC families **A**, **B**: Comparison of DNA sequence chromatogram of an unaffected individual (top) with an affected (bottom). **A**: Patients DNA from KC–01, KC–02 revealed heterozygous T-to-A (c.803 T > A) transition (black arrow) in exon 4 of *VSX1*, resulting in a leucine 268 (CTC) to histidine (CAC) change (p. Leu268His). **B**: Affected individuals from family KC–03 shows a heterozygous T > A (c.751 T > A) nucleotide change in exon 4 of *VSX1*, which altered the serine 251 (TCC) to theronine (ACC) amino acid change (p. Ser251Thr). **C**: Amino acid sequence alignment of the human VSX1 protein (amino acids from 234–274) with other species. The Ser 251 and Leu 268 are shown in red

Direct sequencing analysis of patients from KC families (KC–01_II: 2, KC–02_II:1) (Fig. 1A,B) showed a novel heterozygous c.803 T > A (p. Leu268His) change in exon 4 (Fig. 2A) of the *VSX1* gene. In these families KC was observed in two generations suggesting an autosomal dominant inheritance. This missense substitution had co-segregated in five affected individuals (KC–01_II:1,I:2, KC–02_I:2) with the disease phenotype (Fig. 1A,B). Amino acid conservation analysis revealed that leucine at position 268 was highly conserved in nine vertebrate orthologs and other species (Fig. 2C). This nucleotide change was not present in the 105 normal controls and the unaffected family members.

In another two generation KC family (KC–03) (Fig. 1C), mutation screening of *VSX1* revealed a transition at exon 4 and c.751 T > A was found in three affected (II:2, II:1 I:2) individuals (Fig. 2B). The heterozygous T > A substitution at codon 251 (Ser251Thr) converts a highly conserved amino acid serine (TCC) into threonine (ACC). The unaffected mother (I:1) and 105 controls showed wild type alleles of *VSX1*. None of these identified missense variations have so far been reported in public databases, including NHLBI ESP (http://evs.gs.washington.edu/EVS/), dbSNP (http://www.ncbi.nlm.nih.gov/SNP/) and 1000 Genomes (http://www.1000genomes.org/).

### Clinical findings of the three unrelated KC families

#### Family KC–01

**Fig. 3:**
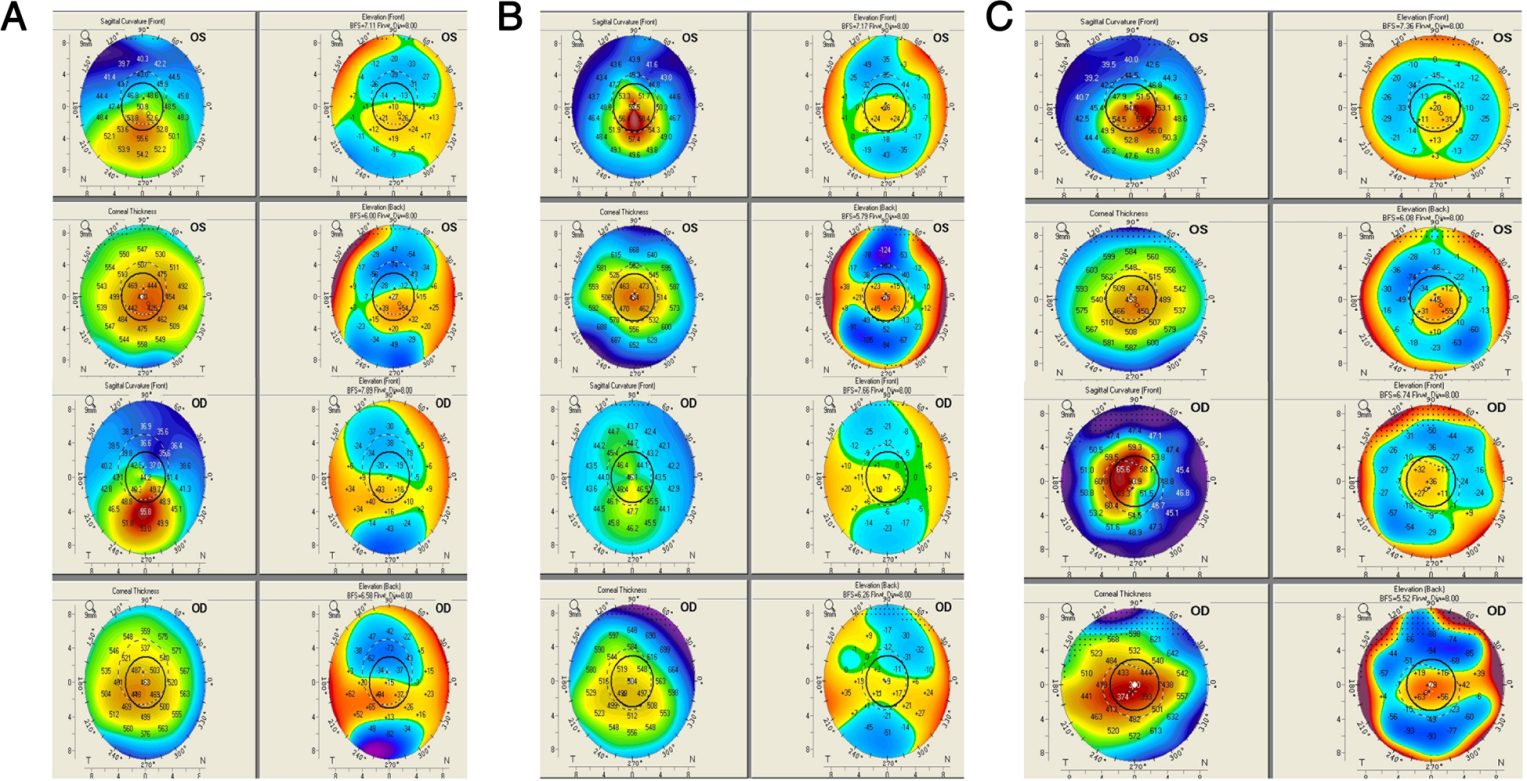
Corneal topography of the KC probands with *VSX1* coding variants. **A**. Pentacam image of patient II:2 from KC–01, both eyes show an area of inferior steepening on the sagittal curvature map with gross inferior-superior asymmetry, more in the right eye. This area of steepening corresponds to areas of abnormal elevation on both the anterior and posterior elevation maps with values suggestive of Keratoconus (KC) with an inferior cone. **B**. In the second family (KC–02), patient II:1 has an area of central steepening on the sagittal curvature map. This area of steepening corresponds to areas of abnormal elevation on both the anterior and posterior elevation maps with values suggestive of KC with a central cone in the left eye (OS); right eye (OD) showing the posterior elevation, suggestive of early KC. **C**. The sagittal curvature on Scheimpflug imaging of patient II:2 from the third family (KC–03) with the left eye showing (OS) an area of central steepening. The anterior and posterior curvature maps show areas of abnormal elevation with values suggestive of KC with a central cone. The corneal thickness map also shows an area of central thinning which is corresponding to the areas of abnormal elevation. The right eye (OD) shows a fairly central area of steepening with features suggestive of advanced KC. There is gross posterior elevation with significant corneal thinning (thinnest pachymetry of 370 μm) in the central 3 mm zone

There are three affected and one unaffected member (Fig. 1A). The proband (II:2) is a 20 year old male with a refraction of–0.5 D spherical and–2.25 D cylinder in his right eye;–1.25 D cylinder in his left eye. Corneal topography (Pentacam, Oculus Inc) revealed grade 1 KC in both eyes with an inferior cone with inferior-superior asymmetry. He had a thinnest pachymetry of 447 μm and 450 μm in his right and left eye respectively (Fig. 3A). He underwent corneal collagen crosslinking in both eyes and was stable at the end of the first year of follow up. The proband’s male sibling (II:1) had a refraction of–1 D spherical with–3 D cylinder in his right eye and–2 D spherical in the left eye. Topography mapping determined grade 1 KC with inferior-superior asymmetry and an inferonasal cone in the right eye with a mean keratometry of 43.6 D and a thinnest pachymetry of 482 μm. He had a normal corneal topography in the left eye. The right eye of the patient I:2 showed grade 1 KC. His refraction in the right eye was–4.25 D spherical with–4.5 D cylinder and in the left eye it was–2.75 D spherical with–3.25 D cylinder. His topography showed a central cone with inferior-superior asymmetry and skewing of 40° in his right eye and normal corneal topography in the left eye.

#### Family KC–02

Consists of four individuals with two having the characteristic features of KC (Fig. 1B). The patient (II:1) is a 16 year old male, with a posterior elevation on topography mapping in the right eye with a grade 2–3 KC in the left eye. Refraction in the right eye was –0.75 D sphere with–1.5 D cylinder and was–8 D sphere with–5 D cylinder in the left eye; mean keratometry was 45.3 D in the right and 53.3 D in the left eye. He had a corneal astigmatism of 2.5 D and 3.6 D and a thinnest pachymetry of 490 μm and 434 μm in the right and left eye respectively (Fig. 3B). The patient underwent corneal collagen crosslinking in the left eye and was stable at follow up two years later. The patient’s father (I:2) had 0.5 D Spherical with–1D cylinder in the right eye and plano refraction in the left eye. His topography evaluation showed normal corneal topography in the right eye while the left eye showed corneal thinning inferiorly, which corresponded with the posterior elevation. Grade 1 KC was noted with a mean keratometry of 43.3 D and pachymetry of 488 μm in the left eye.

#### Family KC–03

This family had one unaffected parent and three individuals with the clinical features of KC (Fig. 1C). The proband (II:2) is an 18 yr old male. Refraction in his right eye was–10 D sphere with–2.25 D cylinder and–3.75 D cylinder in the left eye. The right eye showed advanced KC with a fairly central cone while the left eye showed a grade 2 KC with a mean keratometry of 50.2 D and a thinnest pachymetry of 443 μm (Fig. 3C). He underwent corneal collagen crosslinking in both eyes and was stable on follow up. His sister (II:1) at initial presentation had a normal topography. At the second year follow up (20 years of age), the left eye showed progression to grade 1 keratoconus while the right eye remained stable. Both eyes of the patient I:2 exhibited grade 2 KC; his refraction in the right eye was–6 D spherical with a cylinder of–3.5 D and in the left eye, a sphere of–7.5 D with cylinder of–5 D. His topography scans in both eyes showed inferior steepening with a significant inferior-superior asymmetry.

### *In silico* analysis of the *VSX1* missense variants

*In silico* prediction algorithms of SIFT, Polyphen–2, Provean, and Mutation assessor suggested that the missense change p. Leu268His might negatively affect the function of the coding protein. On the other hand, p. Ser251Thr showed a neutral effect on the functional properties of the protein according to the prediction server results (Table 3). Analysis of amino acid mutation stability for p. Leu268His using the Amino Acid Mutation Stability Prediction Server showed a decrease in the stability of VSX1 protein structure.

**Table 3:** The functional classification and score of *VSX1* variants are predicted by using various bioinformatics tools

c. DNA position	Protein change	Location of protein	Polyphen–2 humDiv	SIFT	Mutation assessor	PROVEAN
c.751 T > A	p. Ser251Thr	CVC domain	Benign (0.9)	Tolerated	Neutral	Neutral
				(0.17)	(0.485)	–2.467
c.803 T > A	p. Leu268His	CVC domain	Possibly damaging for function (1.0)	Deleterious	Functional effect on protein	Deleterious
				(0.05)		–6.831
					(1.905)	

Prediction servers are Polymorphism Phenotyping v2 (PolyPhen–2), Sorting Tolerant From Intolerant (SIFT), Protein Variation Effect Analyzer (PROVEAN), CVC (Chx10/Vsx–1, and ceh–10)

Polyphen–2 scores: 0: benign, 1 possibly damaging for function; 2: Probably damaging for function

SIFT scores: Intolerant or deleterious: score ≤0.05, Tolerant: score >0.05

Mutation Assessor scores: 0–1: no functional effect, 2–3: functional effect on protein function

Provean scores: Cut off threshold = −2.5,

-Variants with a score equal to or below −2.5 are considered “deleterious,”-Variants with a score above −2.5 are considered “neutral

### Haplotype analysis

**Fig. 4:**
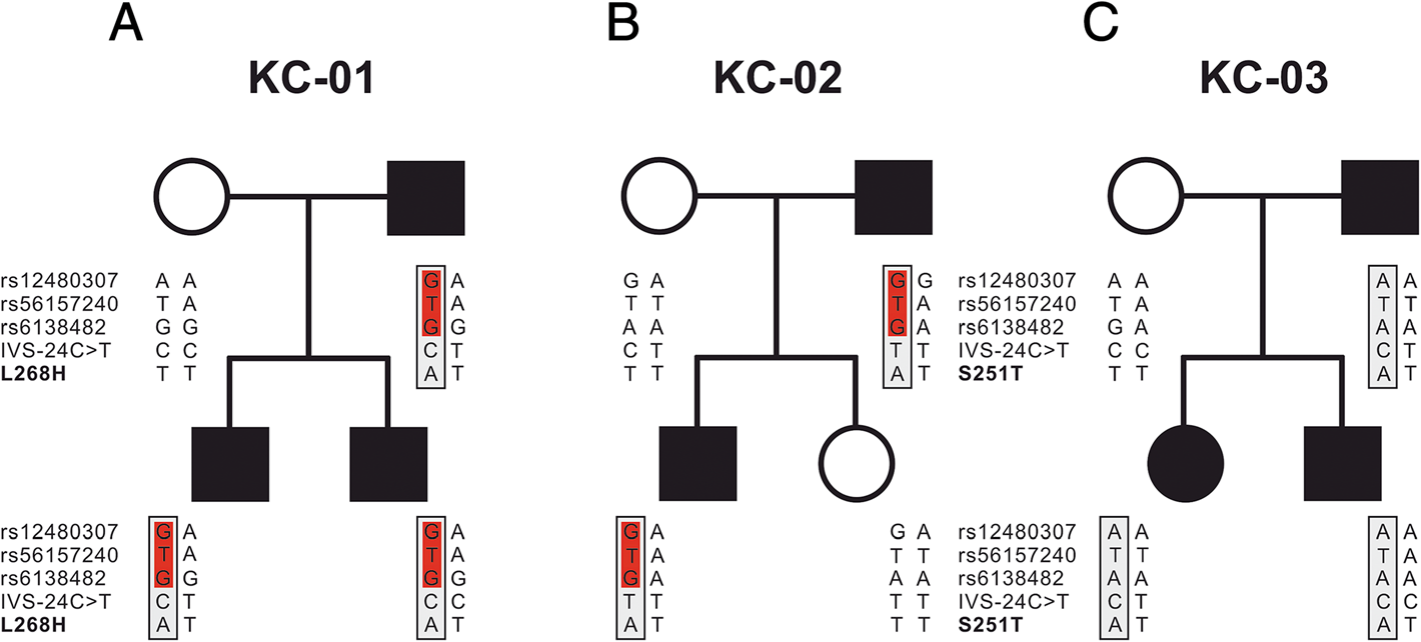
Pedigrees and haplotype analysis of *VSX1* containing L268H and S251T variants in KC families. Legend: **A**,**B**,**C** The haplotype segregating with L268H and S251T are presented on white and gray backgrounds, respectively. Sharing of three common SNPs markers (KC–01,KC–02) families were shown in red

We carried out haplotype analysis to examine whether the missense change, p. Leu268His, was due to a founder effect or was likely to have arisen independently in two unrelated families with KC. The haplotype of affected and unaffected individuals were constructed and compared between families using polymorphic SNPs (rs12480307, rs6138482, rs56157240), and (IVS3–24C > T) markers flanking the *VSX1* gene (Table 4). The haplotype analysis showed that three SNP markers (rs12480307, rs6138482, rs56157240) were shared by five affected individuals (KC–01, KC–02), both families from an endogamous community. (Fig. 4A, B). The affected, and unaffected individuals from the third family (KC–03) showed a different haplotype (Fig. 4C).

**Table 4:** Details of SNP markers used for the haplotype analysis

db SNP ID	Physical position	VSX1 transcript ID	cDNA change	Protein change	Allele frequency^a^	Population frequency^a^
rs12480307	chr20: 25078910	NM_014588	c.546A > G	p.A182A	A : 0.748	A : 75 %
					G : 0.252	G : 25 %
rs56157240	chr20: 25078745	NM_014588	c.627 + 84 T > A	-	T: 0.252	T: 25 %
					A: 0.748	A: 75 %
rs6138482	chr20: 25078806	NM_014588	c.627 + 23G > A	-	G: 0.735	G: 74 %
					A: 0.265	A: 26 %
(IVS3-24C)	chr20: 25078976	NM_014588	c.504-24C > T	-	C:0.999	**Not available**
					T:0.001	

^a^Allele and population frequencies were determined by 1000 Genomes Project Phase 1, HapMap, and ESP for human

## Discussion

Various mutation detection studies have reported that *VSX1* coding variants are associated with KC and PPCD in different ethnic groups (Table 5) [16, 22, 24–26]. In this study, we screened 20 patients of eight unrelated families with KC for mutations in the *VSX1* gene. Among these, five patients from two families had a novel coding variant (p. Leu268His) while another variant (p. Ser251Thr) was identified in a KC family with three affected individuals. The VSX1 consists of a paired-like homeodomain (HD) with a highly conserved CVC domain in the C-terminal, essential for the proliferation, and survival of retinal progenitor cells and bipolar interneurons [32]. The probands from the KC families (KC–01_II: 2, KC–02_II:1) had a heterozygous c.803 T > A nucleotide change associated with clinical features of bilateral KC with a typical sign of Fleisher’s ring. The leucine 268 amino acid residue was located in the C-terminal region of the CVC domain of VSX1 protein. So far only two mutations have been reported in this region in familial KC patients and PPCD [16, 24]. This amino acid change (p. Leu268His) in the CVC domain implied drastic modifications in the physicochemical properties, since leucine is a neutral non-polar amino acid while histidine is a basic-polar residue. It may cause abnormal protein folding that may affect DNA binding properties of the VSX1 transcriptional modulation activity. Moreover, this coding variant (p. Leu268His) leads to the replacement of highly conserved amino acid leucine by histidine in the VSX1 protein, perhaps implicating the functional consequence of this region. Interestingly, CVC domain change (p. His244Arg) has been associated with PPCD along with signs of bipolar cell dysfunction and macular degeneration [33]. According to SIFT, Polyphen-2, Provean, and Mutation assessor, the (p. Leu268His) mutation is found to have a deleterious effect on protein function, attributing a pathogenic nature to this missense mutation in *VSX1*. Furthermore, in our study, this potentially damaging mutation was detected in two families consisting of five affected individuals with a dominant inheritance of KC. This is consistent with previous findings that missense mutations (p. Arg166Trp, p. His244Arg) in *VSX1* were identified in patients with dominant inheritance of KC phenotype [24]. Another study has demonstrated the coding variant p. Gln175His in the homeodomain of VSX1 in an Indian family of KC with incomplete penetrance [25].

**Table 5:** Summary of *VSX1* coding variants identified in patients with KC and PPCD

Coding variants	Clinical significance	Phenotype	Unrelated	Ethnic groups	References
			Controls		
p. Leu17Pro	Pathogenic^**b**^	KC	-	Italian	[26]
p. Leu17Val	Non-pathogenic	KC	+	Korean	[23]
p. Pro58Leu	Pathogenic^**b**c^	KC	-	Caucasian	[22]
p. Asp144Glu	Unknown	PPCD	-	Italian, Ashkenazi Jewish, British, European	[16, 26, 34–37]
p. Leu159Met	Unknown	KC	-	Caucasian	[16, 38]
p. Asn151Ser	Pathogenic^**b**^	KC	-	Korean	[39]
p. Gly160Asp	Non-pathogenic	PPCD	-	Italian, European	[16, 26, 40]
p. Gly160Val	Non-Pathogenic	KC	+	Korean	[23, 39]
p. Val199Leu	Non pathogenic	KC	+	Korean	[23]
p. Arg166Trp	Unknown	KC	+	Caucasian, Iranian	[16, 24]
p. Gln175His	Unknown	KC	-	Indian	[25]
p. Arg217His	Non- Pathogenic	KC	+	Indian, Pakistan, European	[40, 41]
p. Gly239Arg	Pathogenic^**b**c^	KC	-	Italian	[42]
p. His244Arg	Unknown	KC	+	Caucasian, Iranian	[16, 24, 38, 43]
**p. Ser251Thr** ^a^	Unknown	KC	-	Indian	Present study
p. Pro247Arg	Non-pathogenic	KC	+	Italian	[16, 26, 35]
**p. Leu268His** ^a^	Pathogenic^**b**c^	KC	-	Indian	Present study

Coding variants of the *VSX1* gene have been reported in present^a^ and other studies based on original report^b^ and bioinformatics predictions^c^

KC: Keratoconus, PPCD: Posterior polymorphous corneal dystrophy*,* The symbols **“** + ” and “-” represent present and absent, respectively

The probands from KC families showing the variable clinical phenotype, which has been observed in earlier studies as well [34]. On the other hand, it is difficult to establishing a genotype–phenotype correlation in the study subjects due to the presence of inter and even intra-familial clinical variability. Another novel c.751 T > A missense variant was identified in a proband (KC–03_II:2) who exhibited bilateral KC. It is interesting to note that this (p. Ser251Thr) coding variant introduces a missense change that leads to the replacement of highly conserved serine by threonine in the CVC domain of VSX1, probably highlighting the functional importance of this region of the protein. Though serine and threonine have similar properties, threonine is less polar than serine due to the presence of an extra non-polar methyl group. In this context, it may affect the interaction with neighbouring residues that may lead to improper polypeptide folding, thus affecting the protein’s wild-type function. Although this variant was absent in 105 normal controls, *in silico* studies suggest that p. Ser251Thr might be a benign or neutral variant that may not affect the protein function. At this stage, it is difficult to conclude about the pathogenic nature of variants p. Leu268His and p. Ser251Thr. While some previous studies have concluded that missense substitutions in the *VSX1* may or may not be a disease-causing variant [16, 35], others have reported that the mutations were actually non-pathogenic or showed polymorphism [35]. Recent studies have shown that the absence of *VSX1* mutations in a large number of unrelated KC patients suggesting a multiple gene involvement with environmental interaction playing a significant role in the pathogenesis of the disease [36, 37]*.* Haplotype analysis demonstrated a sharing of common SNPs around the missense change (p. Leu268His) in two unrelated KC families, suggesting the possibility of a founder effect, which requires further investigation. The disease causative variants identified in this study were compared to the reported literature of 1–3 % is high (2 probands out of 8, 25 %), probably due to the families belonging to an endogamous community and coincidental selection of study population with high *VSX1* mutations. Further screening of the coding variant (p. Ser251Thr) on a large cohort of familial KC cases may reveal the exact pathological role of the *VSX1* gene. In the eight families who were analysed for *VSX1* mutation screening, we were able to identify a novel missense change (p. Leu268His) in two families and a variant of unknown significance (p. Ser251Thr) in a third family. Screening for other candidate genes including *SOD1, ZEB1, TGFB1, FLG* in KC families could determine the underlying genetic mechanism of the disease in *VSX1* mutation negative patients.

## Conclusions

In summary, we add one novel missense variation in the coding region of VSX1 to the existing repertoire of *VSX1* coding variants observed in Indian patients with the characteristic phenotype of KC. Another variant p. Ser251Thr that was identified may be a benign polymorphism or a variant of unknown significance. Further biophysical studies are necessary to evaluate the precise molecular mechanism of VSX1 caused by this variant. The variation p. Leu268His may be involved in the pathogenesis of KC and therefore help in the genetic counselling of patients and their family.
